# Prevalence, intensity and complications of Microsporidium spores amongst HIV-positive hospital patients in Ilorin, Nigeria

**DOI:** 10.4102/ajlm.v2i1.66

**Published:** 2013-11-26

**Authors:** Amase Nyamngee, Luke D. Edungbola, Olajide O. Agbede, Alakija K. Salami, Charles Nwabuisi, Aliu A. Akanbi, Olatunde O. K. Ibrahim, Muchae Tilahun, Douglas B. Moser

**Affiliations:** 1 Department of Medical Microbiology and Parasitology, University of Ilorin, Nigeria; 2 Department of Medicine, University of Ilorin, Nigeria; 3 Department of Pathology, University of Ilorin, Nigeria; 4 Ethiopian Health and Nutrition Research Institute, Ethiopia; 5 Division of Global HIV/AIDS, U.S Centers for Disease Control and Prevention, USA

## Abstract

**Background:**

Microsporidiasis, which is of great concern for immunocompromised patients, is poorly studied in developing countries.

**Objectives:**

A study was carried out amongst HIV-positive hospital patients and HIV-negative hospital controls in Ilorin, Nigeria, between January 2009 and July 2010 to determine the prevalence and intensity of *Microsporidium* spores and the complications associated with their presence.

**Method:**

Stool samples from 750 HIV-positive patients and 375 HIV-negative patients were studied using the Chromotrope-2R staining technique. Determination of CD4+ count was performed on the Partec Cyflow SL-3 CD4/8 instrument. Intensity of spores was determined by counting the total number of the spores in a 10 µl stained smear of stool. Images were captured with Phenix Microimage Analysis Software and data obtained were analysed using the Statistical Package for the Social Sciences.

**Results:**

The prevalence of *Microsporidium* isolates amongst the HIV-positive hospital patients was significantly higher (42.4%) than amongst the HIV-negative controls (19.2%) (*p* < 0.05). The intensity of microsporidial spores amongst HIV-positive hospital patients was also significantly higher than amongst the controls (*p* < 0.05). However, the difference in the intensity of spores amongst HIV-positive patients who were on antiretroviral therapy (*n* = 411) and those who were not (*n* = 339) was not significant (*p* = 0.236). Microsporidiasis in HIV infection infection was common amongst patients with with low CD4+ counts, diarrhoea, body rashes and cough.

**Conclusion:**

Both the prevalence and intensity of Microsporidiasis are high amongst HIV-positive hospital patients; campaigns to promote awareness, prevention and control are required. Laboratory testing for microsporidia in HIV patients should be performed routinely so as to identify the organism for prompt medical attention.

## Introduction

Microsporidiasis is recognised by the World Health Organization as being a globally-important medical, public health and socio-economic problem.^1,2^ Microsporidia are obligate and opportunistic intracellular spore-forming parasites which belong to the phylum Protozoa.^3,4,5^ Although there are more than 1000 known *Microsporidium* species and as many as 100 genera of the group affecting both vertebrates and invertebrates,^6^ there are only 12 species, belonging to 8 genera, that are known to be pathogenic to humans.^7,8^

The first well-documented human case of Microsporidiasis, where *Encephalitozoon* spp. was detected in a stool sample of a diarrhoea patient, was reported in 1959.^9^ Since then, microsporidia have become increasingly better recognised as opportunistic human pathogens,^10,11,12^ particularly in patients with suppressed immunity, including organ transplant patients and HIV patients.^5,13,14^ Microsporidia can also infect people with competent immune systems, resulting in latent infections.^6,15,16^ In HIV patients, microsporidia have been implicated in intestinal, ocular, pulmonary and renal diseases, and have been shown to cause diarrhea and other adverse health conditions.^17,18,19^

Chronic diarrhoea is common in HIV-positive patients but the enteric pathogens are identified in fewer than 50% of cases.^5,7,8,20,21,22^ The medical and public health implications of chronic diarrhoea in HIV-positive patients are problematic, especially with regard to the management of HIV with antiretroviral therapies.^23,24^

With the detection of more cases of chronic diarrhoea in HIV-positive patients,^4,12,25,26^ *Enterocytozoonbieneusi* (a species of *Microsporidium*) is increasingly suspected to be an aetiological agent, and has also implicated in the excessive morbidity and mortality.^18,26,27,28,29^ In Portugal, Ferreira et al.^30^ reported a 41.2% prevalence of microsporidial diarrhoea in HIV patients, calling for better management of diarrhoea in these patients.

There is a paucity of information on microsporidia, particularly in developing countries where no concerted effort has been made to ascertain the prevalence, intensity, mode of transmission and tissue pathology caused by Microsporidiasis, especially amongst HIV patients. In Nigeria, there is no documented information on human microsporidia and Microsporidiasis. The objective of this study was to determine the prevalence and intensity of *Microsporidium* spores in HIV-positive hospital patients in Ilorin, Nigeria, and to determine the association of Microsporidiasis with other medical conditions amongst these patients.

## Research method and design

### Description of the study area

This work was carried out at the University of Ilorin Teaching Hospital, a tertiary and referral hospital located in Ilorin, Kwara State, Nigeria.

### Study design

A cross-sectional, hospital-based study was designed and implemented so as to determine which pathogenic microsporidia could be detected in both HIV-positive and -negative patients attending the University of Ilorin Teaching Hospital between January 2009 and July 2010. A sample size of 1125 was calculated using Fisher’s formula and based on an estimated 47% prevalence of diarrhoea amongst HIV-positive patients.^5,31^ The sample comprised 750 HIV-positive and 375 HIV-negative patients, matched 2-to-1 for age, sex and other socio-economic variables. Informed consent was obtained from participants before being enrolled for this study. A structured questionnaire was administered to each patient to collect demographic variables and ARV status, as well as information about other concomitant infections. The patients completed the questionnaire before the samples were collected.

### Sampling method

Simple random-sampling by number randomisation was used to select HIV-positive patients for this study. HIV status of patients was determined based on rapid HIV testing kits. HIV-negative controls were selected by number randomisation amongst all possible matches.

### Sample collection and processing

All participants were screened for HIV-status prior to stool collection. The screening and the collection of the stool samples were done by the researcher and two trained laboratory assistants. Three screening methods were used concurrently to test and confirm HIV-sero-status: (1) Antec HIV test (Antec International Ltd, U.K.), (2) Determine HIV 1/2 test (Inverness Medical, Beijing, China) and (3) Uni-Gold HIV test (Trinity Biotech, Ireland). Confirmation of HIV positivity occurred only when all three tests were reactive for HIV antibodies and confirmation for HIV negativity was when all three tests showed no reaction. For the purposes of this study, patients who showed reactions on one or two of the three test kits were retested, but were excluded if the results remained discordant.

**FIGURE 1:**
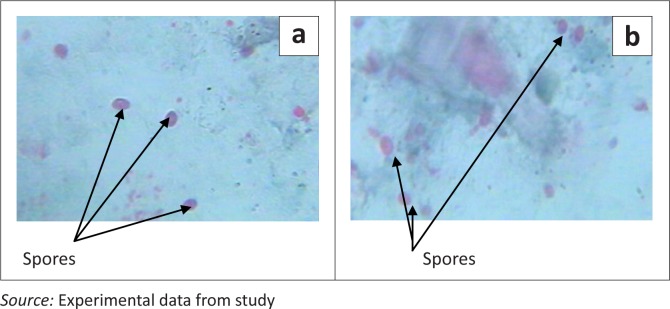
Images of *Microsporidium* spores in stool samples from our study.

Stool specimens were collected in sterile universal bottles from all patients. For the identification of *Microsporidium* spores, unconcentrated formalin-fixed stools were stained using the Chromotrope2R staining method.^5,32^ Each stained smear was examined by light microscope under an oil immersion lens.^5^ The *Microsporidium* photomicrographs and measurement of the spore size were taken according to the manufacturer’s instruction, using Phenix Microimage Analysis Software (PMIAS 3.3), Microscope model ME 100. A positive control slide from the Division of Parasitic Diseases of the Centers for Disease Control and Prevention (CDC), Atlanta, Georgia, reference No. UN3373, was compared against the positive samples in this study. In addition, three positive slides were sent to the CDC in Atlanta for confirmation. The intensity of the spores in the stool samples (Figure 1) was determined by counting the total number of spores in a 10 µl stained smear of stool on a glass slide.^1^ Counting and authentication of the spores was carried out manually by the lead investigator and three independent co-researchers after blinding on HIV sero-status. The average of the 4 results was taken to be the mean intensity of the spores per 10 µl of stool.

### Determination of T-cell count

The CD4+ count was determined using a CD4/8 Cyflow machine (Partec Cyflow SL-3, Germany) according to the manufacturer’s instructions. A 3 ml sample of blood in an Ethylenediaminetetraacetic acid (EDTA) tube was inserted into the machine and the CD4/CD8 count of each individual sample was determined.

### Data analysis

The Statistical Package for the Social Sciences (SPSS version 16.00) was used for data anlysis. Prevalence of infection was given in percentages in line with the variables. Chi-square and 95% confidence interval analysis were the statistical tools used to determine significance at a cut-off value of 95% (*p* = 0.05).

## Results

We enrolled 750 (66.7%) HIV-positive and 375 (33.3%) HIV-negative patients. The prevalence of *Microsporidium* infection amongst the HIV-positive patients was 42.4%, which was significantly higher than the corresponding 19.2% prevalence in the HIV-negative patients (*p* < 0.05; Table 1a). Amongst HIV-positive patients, prevalence of microsporidiasis was significantly lower amongst men than amongst women (39.6% vs. 44.9%, *p* < 0.05). Differences in prevalence between men and women amongst HIV-negative patients (19.9% vs. 18.6%) and amongst all patients (33.1% vs. 36.1%) did not reach statistical significance (*p* > 0.05) (Table 1b).

**TABLE 1a:** Prevalence of Microsporidiasis by age amongst HIV-positive and -negative patients (*n* = 1125).

Age (years)	HIV-positive patients		HIV-negative patients	
	No. Examined	No. (%) +ve	No. Examined	No. (%) +ve
≤ 1	11	3 (27.3)	6	2 (33.3)
2–11	53	14 (26.4)	28	0 (0.00)
12–21	175	56 (32.0)	108	14 (13.0)
22–31	238	76 (31.9)	113	20 (17.7)
32–41	152	89 (58.6)	64	27 (42.2)
42–51	99	63 (63.4)	47	5 (10.6)
52–61	22	17 (77.3)	9	4 (44.4)
**Total**	**750**	**318 (42.4)**	**375**	**72 (19.2)**

+ve; Positive for Microsporidiasis

**TABLE 1b:** Prevalence of Microsporidiasis amongst HIV-positive and -negative patients by gender (*n* = 1125).

Infection status	Male		Female		Total
	No. Examined	No. (%) +ve	No. Examined	No. (%) +ve	No. (%) +ve
HIV-positive patients (*n* = 750)	356	141 (39.6)	394	177 (44.9)	218 (42.4)
HIV-negative patients (*n* = 375)	176	35 (19.9)	199	37 (18.6)	72 (19.2)
**Total (*n* = 1125)**	**532**	**176 (33.1)**	**593**	**214 (36.1)**	**390 (34.7)**

+ve; Positive for Microsporidiasis

The prevalence amongst HIV-positive patients who had been on ARVs for between 3–18 months was similar to that amongst patients not on ARVs (67.6% vs. 68.2%, *p* >0.05) (Table 2). All HIV-positive patients aged 42–61 who were not on ARVs had Microsporidiasis; their counterparts who were on ARVs also had a high prevalence. The prevalence of Microsporidiasis increased with age amongst both groups.

**TABLE 2:** Prevalence of Microsporidiasis amongst HIV-positive patients by age and ARV status (*n* = 750).

Age (Years)	On ARVs		Not on ARVs	
	No. % Examined	No. (%) +ve	No. (%) Examined	No. (%) +ve
≤ 1	0 (0.0)	0 (0.0)	11 (100.0)	3 (27.3)
2–11	4 (7.5)	1 (25.0)	49 (92.5)	13 (26.5)
12–21	82 (46.9)	38 (46.3)	93 (53.1)	43 (46.2)
22–31	148 (62.2)	90 (60.8)	90 (37.8)	55 (61.1)
32–41	102 (67.1)	92 (90.2)	50 (32.9)	48 (96.0)
42–51	61 (61.6)	46 (75.4)	38 (36.6)	38 (100.0)
52–61	14 (63.6)	11 (78.6)	8 (22.7)	8 (100.0)
**Total**	**411 (54.8)**	**278 (67.6)**	**339 (45.2)**	**217 (68.2)**

+ve; positive for Microsporidiasis, ARV; Antiretroviral therapy

The mean intensity of *Microsporidium* spores per 10 µl of stool amongst the HIV-positive patients who were on ARVs showed no significant difference when compared with those who were not on ARVs (Table 3). The 52–61 age group demonstrated the highest intensity of *Microsporidium* spores (mean±standard deviation: 277±94.0) amongst HIV-positive patients who were on ARVs. Patients not on ARVs had the highest intensity of spores (234 ± 88.0) in the 32–41 age group. The difference in intensity amongst those patients on ARVs and those not on ARVs in relation to age was not statistically significant (*p* > 0.05). In both groups, intensity of *Microsporidium* spores was inversely associated with CD4+ count; age groups with low CD4+ had high intensity of *Microsporidium* (Table 3).

**TABLE 3:** Intensity of *Microsporidium* spores in relation to age, CD4+ count and antiretroviral therapy status amongst HIV-positive patients (*n* = 750).

Age	Number of Patients		Mean ± SD Intensity of Spores		Mean ± SD CD4+ Cell Count	
	On ARVs (*n* = 411)	Not on ARVs (*n* = 339)	On ARVs	Not on ARVs	On ARVs	Not on ARVs
≤ 1	0	11	N/A	16 ± 5.2	N/A	891 ± 304.4
2–11	4	14	106 ± 48.0	140 ± 57.0	916 ± 311	544 ± 198.3
12–21	82	93	128 ± 51.0	164 ± 59.0	371 ± 92	263 ± 72.6
22–31	148	90	194 ± 58.0	201 ± 62.0	212 ± 81	145 ± 58.4
32–41	102	50	234 ± 88.0	244 ± 82.0	124 ± 53	112 ± 21.1
42–51	61	38	203 ± 61.0	241 ± 78.0	217 ± 83	114 ± 22.3
52–61	14	8	211 ± 66.0	277 ± 94.0	131 ± 56	11 ± 3.3

SD; standard deviation, ARV; antiretroviral therapy

Amongst HIV-positive patients, those with diarrhoea had the highest prevalence of Microsporidiasis amongst all those with a single condition (95.6%) (Table 4). This was followed closely by those with body rashes (91.7%). Amongst those with multiple conditions, those with diarrhoea and body rashes (with and without cough) had 100% prevalence of Microsporadiasis.

**TABLE 4:** Association of Microsporidiasis with other disease conditions in HIV-positive cases.

S/No.	Disease Conditions	No examined in each case (*n*)	No. (%) +ve for microsporidia	CD4+ range for microsporidia +ve samples	Mean±SD of CD4+ for microsporidia +ve samples
1	Pregnancy	28	21 (75.0)	234	127 ± 76
2	Cough	216	108 (50.0)	216	142 ± 84
3	Tumour cells	9	4 (44.4)	132	96 ± 50
4	Diarrhoea	318	304 (95.6)	120	87 ± 48
5	Body rashes	216	198 (91.7)	103	74 ± 36
6	Fever	272	192 (70.7)	122	88 ± 41
7	General body weakness	192	93 (48.4)	243	92 ± 36
8	Diarrhoea and cough	296	228 (77.0)	181	86 ± 42
9	Fever and diarrhoea	396	209 (52.8)	172	76 ± 28
10	Diarrhoea and body rashes	298	298 (100.0)	114	69 ± 23
11	Body rashes and cough	273	191 (70.0)	111	62 ± 21
12	Cough, diarrhoea and body rashes	302	302 (100.0)	97	11 ± 6

+ve; Positive

## Ethical considerations

### Ethical clearance

Ethical clearance was sought and obtained from the Ethical Review Committee of the University of Ilorin Teaching Hospital with reference number, UITH/CAT/189/817.

### Informed consent

The purpose of this study was explained in detail to all subjects during the study. Participation was voluntary and the confidentiality of information given for the purpose of this study was guaranteed.

All subjects were also apprised of the benefits of knowing their HIV status, CD4+ counts, and medical complications.

### Data protection

All specimen collected were preserved according to international standards and kept under iced Hydrogen refrigeration in the data storage chamber of the University of Ilorin Teaching Hospital.

## Trustworthiness

Independent researchers confirmed and counted the spores to address the issue of credibility and the average of the four counts was recorded. We also obtained a control slide of the organism from the CDC in order to ensure that the organism was identified correctly. To address the issues of dependability and confirmability, we relied on an average independent count and the control slide.

## Discussion

Our findings revealed a high prevalence (42.4%) and significant intensity of Microsporidiasis amongst the HIV-positive patients, thus complicating morbidity and mortality in areas where HIV is common. The infection was detected across genders and age groups indicating that microsporidia are ubiquitous as suggested previously.^6,16^ Whilst prevalence increased with age amongst the HIV-positive patients, there was was not a clear trend amongst the HIV-negative controls. We observed that the intensity of spores was highest amongst the HIV-positive patients, especially in the very old and in those with very low (< 10) CD4+ counts, suggesting a relationship with the level of CD4+ counts in HIV-positive patients^19^. Previous studies have reported a high intensity of microsporidia in HIV-positive patients due to their low immune status, which created a condition for the rapid replication of *Microsporidium* spores^12^.

Amongst the HIV-positive patients, it was observed that 95.6% of those reporting with diarrhoea were positive for *Microsporidium*, which may implicate microsporidia as an aetiologic agent in the diarrhoea that is seen in these immunocompromised patients. This is in line with previous suggestions concerning the need for more investigations of the aetiologic agent of this diarrhoea amongst HIV-postive patients.^5,18^ A 100% prevalence of *Microsporidium* spores was seen amongst HIV-positive patients who had both diarrhoea and body rashes (with or without cough). Other conditions associated with *Microsporidium* were body rashes only (91.7%), diarrhoea with cough (77%) and body rashes with cough (70.0%), stimulating interest about the involvement of Microsporidiasis in other complications amongst HIV patients. This is contrast with the findings of Nkinin et al.^16^ who reported an unexpectedly high prevalence (67.5%) of Microsporidiasis in 126 immunocompetent patients in Cameroon in 2007, but found no clinical manifestations in these individuals.

Presently, there is no effective treatment for treating Microsporidiasis which has made control difficult, even in areas where there is knowledge regarding *Microsporidium* infections in humans.^23,24^

## Limitations of the study

This study is subject to several limitations. First, measuring the intensity of spores in stool samples is somewhat subjective and variable; to reduce these issues, we had 4 experts independently count spores and averaged the results. Second, the study was hospital-based, limiting our inference to the general population. Third, irregular power supply may have adversely affected some of our samples; this was reduced through the provision of an alternative power supply.

### Recommendations

We recommend that routine laboratory screening be performed for microsporidia in the hospital setting and that positive isolates be made notifiable. More studies on the aetiology of Microsporidiasis (under light- and electron microscopy) should be undertaken, which should include histopathology study of different organs at autopsy.

## Conclusion

We found a high prevalence and intensity of microsporidia amongst HIV-positive patients, especially amongst those with diarrhoea, body rashes and/or cough. These findings should provide base-line information for further studies and provoke interest in investigations of microsporidia that aim at determining the mode of infection, pathogenicity and treatment as well as prevention and control.

Health education campaigns to promote awareness, prevention and control of Microsporidiasis should be conducted regularly, particularly amongst HIV-positive patients as well as medical, public health and laboratory personnel. Positive results for microsporidia obtained through routine laboratory screening in hospitals should be notifiable. Finally, based on the prevalence and implications of microsporidial infection as observed in this study, there is a need for the development of an ideal drug for the treatment of Microsporidiasis.
